# Dental practice closure during the first wave of COVID-19 and associated professional, practice and structural determinants: a multi-country survey

**DOI:** 10.1186/s12903-021-01601-4

**Published:** 2021-05-07

**Authors:** Hams Abdelrahman, Sara Atteya, Merna Ihab, Myat Nyan, Diah A Maharani, Anton Rahardjo, Mohammed Shaath, Khalid Aboalshamat, Syeda Butool, Anas Shamala, Lubna Baig, Maha El Tantawi

**Affiliations:** 1 Department of Pediatric Dentistry and Dental Public Health, Faculty of Dentistry, Alexandria University, Champlion St., Azarita, Alexandria, 21527 Egypt; 2 Department of Prosthodontics, University of Dental Medicine, Mandalay, Myanmar; 3 Department of Preventive and Public Health Dentistry, Faculty of Dentistry, Universitas Indonesia, Depok, Indonesia; 4 ParkHouse Dental Group, Manchester, UK; 5 Dental Public Health Division, Preventative Dentistry Department, College of Dentistry, Umm Al-Qura University, Makkah, Saudi Arabia; 6 Department of Conservative and Endodontics, National Institute of Medical Sciences University and Research, Jaipur, Rajasthan India; 7 Department of Biological and Preventive Sciences, College of Dentistry, University of Science and Technology, Sanaa, Yemen; 8 APPNA Institute of Public Health, Jinnah Sind Medical University, Karachi, Pakistan

**Keywords:** COVID-19, Dentists, Dental clinics, Private practice, Hospital bed capacity, Fear

## Abstract

**Background:**

The coronavirus outbreak (COVID-19) in China has influenced every aspect of life worldwide. Given the unique characteristics of the dental setting, the risk of cross-infection between dental practitioners and patients is high in the absence of adequate protective measures, and dentists may develop severe anxiety in relation to the current pandemic. The limited provision of services and widespread closure of dental practices have raised concerns among dental professionals about the financial impact. The present study assessed the frequency of dental practice closure during the pandemic’s first wave in several countries and whether closures and their associated factors differ between the private and non-private sectors.

**Methods:**

An electronic cross-sectional survey questionnaire was sent to dentists in several countries, from April to May 2020. The survey assessed professional, practice related and country-level structural factors elucidating the reason for practice closure. Multilevel logistic regression was used to assess the association between practice closure and these factors, and differences were evaluated by sector type.

**Results:**

Dentists from 29 countries (n = 3243) participated in this study. Most of the participants (75.9%) reported practice closure with significantly higher percentage in the private sector than the non-private sector. Greater pandemic-related fears were associated with a significantly higher likelihood of practice closure in the private (odds ratio [OR] = 1.54, 95% confidence interval [CI] 1.24, 1.92) and non-private (OR = 1.38, 95% CI 1.04, 1.82) sectors. Dentists in non-private rural areas (OR = 0.58, 95% CI 0.42, 0.81), and those in hospitals (overall OR = 0.60, 95% CI 0.36, 0.99) reported a low likelihood of closure. A high likelihood of closure was reported by dentists in the academia (OR = 2.13, 95% CI 1.23, 3.71). More hospital beds at the country-level were associated with a lower likelihood of closure in the non-private sector (OR = 0.65, 95% CI 0.46, 0.91). Private- sector dentists in high- income countries (HICs) reported fewer closures than those in non-HICs (OR = 0.55, 95% CI 0.15, 1.93).

**Conclusions:**

Most dentists reported practice closure because of COVID-19, and greater impacts were reported in the private sector than in the non-private sector. Closure was associated with professional, practice, and country-levels factors.

**Supplementary Information:**

The online version contains supplementary material available at 10.1186/s12903-021-01601-4.

## Background

On January 8, 2020, the Chinese Center for Disease Control and Prevention declared that a coronavirus causes COVID-19 [1]. Since then, COVID-19 has become a major public health problem worldwide and caused various changes in all aspects of life [2]. As of April 17, 2021, COVID-19 has been reported in 47 countries in Africa, 15 countries in the Western Pacific region, 53 countries in Europe, 10 countries in Southeast Asia, 21 countries in the Eastern Mediterranean region, and 35 countries in the region of Americas with a total of 139,501,934 million laboratory-confirmed cases, about 2,992,193 million deaths and 751,452,536 vaccine doses have been administered [3].

COVID-19 transmission occurs interpersonally via respiratory droplets, inhalation, ingestion, or direct mucosal contact with saliva droplets [4]. The virus can survive on hands, objects, or surfaces previously exposed to infected saliva [4]. The current evidence suggests that the virus mainly spreads by respiratory droplets, among individuals in close contact with each other (less than 1 m apart) [5].

The incubation period of the virus causing COVID-19 has been reported to range from 2 to 14 days [1, 6]. The clinical manifestations of the disease include mild, flu-like symptoms, such as fever (98%) and dry cough (76%). Patients may develop respiratory and multiple organ failure leading to death. Lymphocytopenia may be observed, and chest CT examination usually shows a ground-glass appearance of the lungs [7].

The clinical management and treatment of COVID- 19 still rely on the symptomatic management of patients. Treatment is achieved by controlling secondary infection through administration of broad-spectrum antibiotics and organ support in intensive care units.

On March 5, 2021, the National Institute of Health (NIH) published recommendations for using Immunomodulators as Tocilizumab and Anti-SARS-CoV-2 Monoclonal Antibodies as Bamlanivimab. Antiviral drugs as Redmisivir and Corticosteroids as Dexamethasone were suggested by the COVID-19 Treatment Guidelines Panel as the latest information regarding the treatment of COVID-19. The choice of treatment line is based on the patient’s clinical presentation [8].

In addition, several vaccines are already available in the market [9], with inter-country variations in availability [10] although, the induction of herd immunity and the slowing down of the spread of the virus is still to be achieved [11]. In addition to those in the market, other types of vaccines are also being tested. The most Promising COVID-19 vaccines are Pfizer-BioNTech and Moderna’s achieving 90% effectiveness followed by Johnson & Johnson's, Astrazeneca and Novavax [12].

Healthcare professionals are at the forefront of the fight against COVID-19 with the number of infected cases and fatalities varying between countries. Given the unique nature of the dental practice, the risk of cross-infection is higher among dentists than other healthcare professionals in the absence of protective measures [13, 14], especially since the virus has been identified in the saliva of infected patients [15]. These factors directly contribute to the higher level of COVID-19-related fear and anxiety among dentists compared to the general public [16].

During the first wave of the pandemic, routine dental services were restricted due to shortages of personal protective equipment (PPE), to help flatten the curve, and protect patients and dental personnel against infection. Urgent care was delivered using PPE, and additional precautions, including taking patients’ recent travel history, recording patients’ body temperature, using 1% hydrogen peroxide as a pre-procedural mouth rinse, using a rubber dam and high-volume suction, and frequent cleaning and disinfection of public contact areas such as door handles, chairs and washrooms, were implemented [17]. The reduced availability of dental care has increased demands on already- burdened hospital emergency departments [19], with potential adverse impacts on oral health and quality of life. Moreover, the closure of dental practices has raised concerns among dentists about the financial implications of this measure [20]. Reports show that the economy of the dental and other healthcare sectors was virtually halted because of the pandemic [20–22].

The disruption of dental care delivery that occurred at a large scale during the first wave might have had great effects on dentists, other healthcare personnel, and the general public [22]. Thus, assessing the extent of dental practice closure during the first wave and its determinants is important to help mitigate its impact and plan supportive measures. It also offers lessons to develop strategies during the third wave of the pandemic and help plan the future of the dental practice during the pandemic.

The present study aimed to assess the extent of dental practice closure as reported by dentists from different countries during the first wave of the pandemic, the factors associated with these closures, and whether closures and their associated factors differed between the private and non-private sectors. The null hypothesis of the study was that dental practice closure during the first wave was neither affected by professional (COVID-19 knowledge and fears), practice- related (private or non-private sectors, in urban or rural areas and solo or group practices), or country- level (country-level number of hospital beds representing a country’s ability to mobilize resources to control the disease and country-level income indicating the potentially available financial support for dentists in case of practice closure) attributes.

## Methods

### Design

A cross-sectional, multi-country, electronic survey questionnaire was conducted from April to May 2020 during the first wave of the pandemic. The Ethics Review Committee of the Faculty of Dentistry, Alexandria University approved the study including how the consent was obtained (IRB No. 00010556-IORG 0008839). The study was carried out according to the principles of the Declaration of Helsinki.

### Participants and sample size

The target population was dental practitioners across the globe. The study recruited specialists and non-specialists practicing in various health care and dental academic sectors during the study period. Dental students and interns were excluded. The sample size was calculated [23] based on previous estimates: Zhang et al. [24] reported that the percent change in healthcare services utilization post epidemic ranged from 12 to 18% around the mean hypothesized to be 50%. Thus, in a best-case scenario, participants may still follow the pre-pandemic estimates (50%) or their utilization may have dropped to a worst case scenario of 12%-18%. Based on comparison of proportions, we calculated [23] that between 254 and 568 participants would be needed. A larger number of participants were eventually included because of the multi-country nature of this study and the difficulty of limiting the number of participants once the survey link was posted on social media.

### Questionnaire design and pilot testing

The questionnaire (Additional file 1) was designed on the basis of a previously published tool [25] and updated using information obtained from the websites of the World Health Organization (WHO), American Dental Association, and the American Center for Disease Control. To assess its content validity, two dental academics was given a content validation form and asked to judge the degree of relevance of each item using a 4 point ordinal scale: (1) not relevant, (2) somewhat relevant, (3) quite relevant, and (4) highly relevant. The content validity at item level (CVI-I) was calculated by dividing the number of experts giving a score of 3 to 4 for each relevant item, by the total number of experts. Overall, a CVI-I score of 0.93 was obtained, which was considered appropriate [26].

To examine face validity, 15 dentists participated in the pilot testing of the questionnaire to assess its clarity, and whether any questions were missing, irrelevant and/or confusing. The survey was translated into German and Italian by two independent bilingual dentists (Additional file 3). Using back translation, the translated questionnaires were compared with the original English version to identify discrepancies and resolve vagueness. The German version was pilot tested by 14 dentists, and the Italian version was tested by 11 dentists. The Pilot testing results were not included in the analysis.

At the beginning of the survey, a short introduction described the study purpose and assured the participants of the confidentiality of their responses. The questionnaire included 31 close-ended questions and was divided into three sections. Section 1 included questions related to the sociodemographic and practice-related characteristics of the participants, including their age, gender, country of practice, specialty, area of practice (rural or urban), and type of practice (private, governmental, or academic sector; solo, group, or hospital practice). Section 2 included eight items assessing dentists’ fears regarding COVID-19; the responses in this section were scored using a five-point Likert scale ranging from strongly agree (code 5) to strongly disagree (code 1). Section 3 consisted of 15 questions assessing knowledge about measures to control the transmission of COVID-19; the items in this section could be answered by yes, no, or do not know. The last question in the form asked whether the participants’ dental practice was closed at the time of the survey.

### Data collection

A link to the electronic survey was created using the online survey platform "Survey Monkey''. Respondents were allowed to change their responses prior to submission, and no duplicate entries were allowed. The questionnaire could be completed within 5–7 min. A call for collaborators was sent via Research Gate and by email to personal contacts. Interested researchers received further information about the study proposal, sampling strategy, timeline and responsibilities. Researchers willing to collaborate were invited to the study team and the data they collected were included in the study. The link was sent to collaborators, and convenience and snowball sampling were applied to promote the link via dentists’ groups on Facebook, Instagram, LinkedIn, Twitter and WhatsApp. Examples of these webpages included those of dental syndicates/ associations, webpages for postgraduate students in different universities/ countries, online forums such as “World of Dentistry”, “Style Italiano” and others. Participants were also asked to share the survey with their dental contacts.

### Statistical analysis

The overall score of fears and threats was averaged from the scored of the eight items in Sect. 2 and ranged from 1 to 5. The overall knowledge score was obtained by assigning one point for each correct answer to the questions in Sect. 3 and then adding the points of the 15 items; the total score for this section ranged from 0 to 15. Cronbach's alpha was used to assess the internal consistency of items related to COVID-19 fears and knowledge. The Cronbach’s alpha values ranges between 0 and 1 with 0 denoting complete inconsistency/ disagreement and 1 denoting complete agreement/ consistency. Values above 0.60 are considered acceptable [27, 28].

Multilevel logistic regression analysis was used to assess the association between the dependent variable (i.e., practice closure with yes/ no responses) and the independent variables that were introduced as fixed effects. These were level 1 dentist and practice factors obtained from the survey, in addition to level 2 country-level structural factors. The latter factors included the country-level number of beds per 1000 population obtained from the World Bank Databank [29]. In the absence of multi-country data regarding the number of intensive care units needed to care for patients with COVID-19 complications, we used the number of beds per 1,000 population as an indicator of the availability of inpatient services [30]. Country-level factors also included income level based on the World Bank classification of high- income countries (HICs) with a gross national income (GNI) > 12,375 US$, upper middle-income countries (UMICs) with GNI between 3,996 and 12,375 US$, lower middle-income countries (LMICs) with GNI between 1,026 and 3,995 US$ and low-income countries (LICs) with GNI < 1026 US$ [31]. These levels were recoded into HICs and non-HICs. Country was included as a random effect factor. Robust estimation was used to address violations of model assumptions. A model was developed for the whole sample, and two additional models were developed for participants working in the private sector and those working in the non-private sector. Odds ratios (ORs) and 95% confidence intervals (CIs) were calculated. Effect modification by sector (i.e., private and non-private) was assessed, and p values were computed for interaction. Significance level was set at 5%, and SPSS version 23.0 [32] was used for statistical analysis.

## Results

A total of 3,243 dentists from 29 countries responded to the survey (Additional file 2). Among the respondents, 49.2% were 20–30 years old, 56.8% were females and 70.6% were specialists. Moreover, 65.6% of the respondents worked in the private sector, 52.3% were in group practice, and 81.8% worked in urban locations. The mean (SD) number of beds/ 1,000 population was 1.70 (0.99), and 71.7% of the dentists were from non-HICs. Most of the participants (75.9%) reported that their practices were closed because of the pandemic (Table 1). Dentists working in the private sector were significantly more likely to report practice closure than those working in the non- private sector reported practice closure (78.3% and 71.3%, *P* < 0.0001).

**Table 1:** Distribution of personal, professional and practice and structural factors among the study participants (n = 3243)

Factors	N (%)
*Personal*	
Age	
20–30	1597 (49.2)
31–40	986 (30.4)
41–50	420 (13)
51–60	193 (6.0)
61+	47 (1.4)
Gender	
Males	1401 (43.2)
Females	1842 (56.8)
*Professional and Practice*	
Specialty	
General practitioners	2290 (70.6)
Specialists	953 (29.4)
Practice nature	
Private sector	2123 (65.6)
Governmental clinic	1263 (38.9)
Clinics in an academic institution	528 (16.3)
Practice type	
Solo practice	1129 (34.8)
Group practice	1697 (52.3)
Hospital	998 (30.8)
Practice setting	
Urban location	2653 (81.8)
Rural location	590 (18.2)
*Structural*	
Number of beds/ 1000 population	
Mean (SD)	1.70 (0.99)
High income countries	
Yes	917 (28.3%)
No	2326 (71.7%)
Practice closed because of the pandemic	
Yes	2461 (75.9)
No	782 (24.1)

**Fig. 1:**
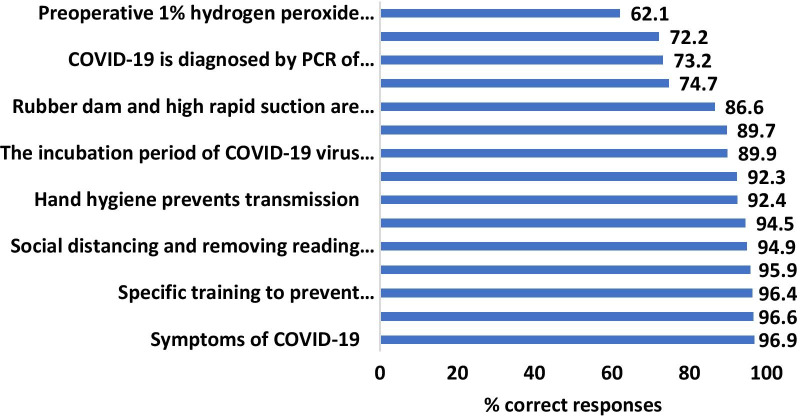
Correct responses regarding knowledge about COVID-19 pandemic

Table 2 shows the underlying causes of fear due to COVID-19. The greatest fears were of family members catching infection (mean = 4.36) and the high infection risk among healthcare personnel (mean = 4.21). Cronbach’s alpha for the internal consistency of fear related items was 0.70. The mean fear score was 4.14 out of 5 (SD = 0.54). Figure 1 shows that > 95% of the dentists had correct knowledge about COVID-19 symptoms, transmission through respiratory secretions, specific training being needed to prevent infection and precautions needed against droplet, contact and airborne infections. Cronbach’s alpha for the internal consistency of knowledge- related items was 0.62, and the mean knowledge score was 13.08 out of 15 (SD = 1.89).

**Table 2:** Levels of fears and perceived threat because of the COVID-19 pandemic reported by participating dentists

Item	Mean (SD)
I am afraid of working in places where patients suspected of COVID-19 infection are treated	4.08 (0.99)
I am afraid of providing dental care for patients infected with/ suspected of COVID-19	4.14 (1.00)
In spite of PPE and infection prevention precautions, the risk of COVID-19 infection is high among health care personnel	4.21 (0.89)
Equipment and facilities required to protect HCP from COVID-19 infection are not adequately provided in healthcare facilities	4.13 (0.92)
Healthcare personnel should be paid more when treating patients infected with/ suspected of COVID-19 infection	3.91 (1.13)
I am afraid that a family member may be affected by COVID-19 infection	4.36 (0.90)
I am worried that my patients will not be receiving adequate care because of the outbreak	4.08 (0.79)
I am worried that my practice income would be affected because of the outbreak	4.16 (0.92)

Table 3 shows the factors associated with practice closure and how they differ between the private and non-private sectors. In terms of dentist factors, greater fear was associated with a significantly higher likelihood of reporting closure by dentists in the private sector (OR 154, 95% CI 1.24, 1.92) than by those in the private sector (OR 1.38, 95% CI 1.04, 1.82), with no significant difference between groups (*P* = 0.21). There was a significant difference between private sector and non-private sector dentists (*P* = 0.03) in the association between practice closure and specialization. In the private sector, there was a significantly greater likelihood of reporting closure by general practitioners than specialists (OR 1.38, 95% CI 1.04, 1.95). This association was not statistically significant in the non-private sector (OR 1.08, 95% CI 0.77, 1.53).

**Table 3:** Factors associated with practice closure because of COVID-19 in multilevel regression in private and non-private sectors

Factors	Private sector N = 2123	Non-private sector N = 1120	All participants N = 3243	*P* of interaction
	OR (95% CI)			
Professional factors				
Specialty				
GP	1.38 (1.04, 1.85)*	1.08 (0.77, 1.53)	1.14 (0.82, 1.59)	0.03*
Specialist	Reference category			
Fear score	1.54 (1.24, 1.92)*	1.38 (1.04, 1.82)*	1.28 (0.97, 1.67)	0.21
Knowledge score	1.05 (0.98, 1.12)	1.03 (0.96, 1.11)	1.01 (0.94, 1.09)	0.21
Practice factors				
Clinic in an academic institution	1.37 (0.93, 2.03)	1.81 (1.04, 3.15)*	2.13 (1.23, 3.71)*	0.13
Clinic in non-academic institution	Reference category			
Solo practice	1.45 (1.00, 2.10)	1.14 (0.66, 1.97)	1.13 (0.65, 1.94)	0.50
Non-solo practice	Reference category			
Group practice	0.88 (0.60, 1.29)	0.79 (0.49, 1.28)	0.80 (0.49, 1.29)	0.85
Non-group practice	Reference category			
Hospital setting	0.55 (0.41, 0.75)*	0.58 (0.35, 0.97)*	0.60 (0.36, 0.99)*	0.64
Non- hospital setting	Reference category			
Setting				
Rural	1.29 (0.91, 1.82)	0.58 (0.42, 0.81)*	0.58 (0.42, 0.81)*	0.001*
Urban	Reference category			
Structural factors				
Beds/1000 population	1.01 (0.72, 1.41)	0.65 (0.46, 0.91)*	0.94 (0.65, 1.37)	0.96
Income				
HICs	0.55 (0.15, 1.93)	2.00 (0.60, 6.69)	0.60 (0.19, 1.90)	0.64
Non-HICs	Reference category			

The models are adjusted for gender and age

Bold values indicate significant difference, *Statistically significant at *p* value ≤ 0.05

*OR* odds ratio, *CI* confidence interval, *GP* general practitioner, *HICs* high income countries

In terms of practice- related factors, working in hospitals was associated with a significantly lower likelihood of closure in the private (OR 0.55, 95% CI 0.41, 0.75) and non-private sectors (OR 0.58, 95% CI 0.35, 0.97); no significant difference was found between groups (*P* = 0.64). In addition, in both groups alike (*P* = 0.13), there was a significantly higher likelihood of reporting closure by dentists working in the academia (OR 2.13, 95% CI 1.23, 3.71). On the other hand, there was a significant difference between private and non-private sectors (*P* = 0.001) in the association between practice closure and practice location (i.e., urban vs. rural). Dentists in the private sector reported non significantly higher likelihood of practice closure in rural than urban locations (OR 1.29, 95% CI 0.91, 1.82). By contrast, dentists in the non-private sector reported significantly lower likelihood of practice closure in rural than in urban areas (OR 0.58, 95% CI 0.42, 0.81). Overall, dentists in solo practice were more likely to report practice closure than those not in solo practice (OR 1.13, 95% CI 0.65, 1.94), with no significant difference between groups (*P* = 0.50). On the other hand, dentists in group practice were less likely to report practice closure than those not in group practice (OR 0.80, 95% CI 0.49, 1.29), with no significant difference between groups (*P* = 0.85).

Regarding country-level structural factors, there were no significant differences between private and non-private sectors in the association between practice closure and the number of hospital beds (*P* = 0.96) or country high income (*P* = 0.64). More hospital beds were associated with a significantly lower likelihood of practice closure among dentists in the non-private sector (OR 0.65, 95% CI 0.46, 0.91). Dentists in the private sector who practiced in HICs were non-significantly less likely to report practice closure than those from non-HICs (OR 0.55, 95% CI 0.15, 1.93).

## Discussion

The results of this study showed that, from April to May 2020, 75.9% of the dentists reported practice closure with a higher percentage in the private than the non-private private sector. Dentists in the private sector, who were general practitioners, in solo practice, in rural areas, and with greater COVID-19 fears were also more likely to report practice closure. Country-level determinants were associated with practice closure. For example, better-prepared healthcare systems were associated with fewer closures in the non-private sector, and private-sector practices in richer countries were less likely to close than those in less affluent countries. Thus, the null hypothesis was rejected.

This study provides compelling evidence of the impact of the first wave of COVID-19 on dental practice closures, which jeopardized the provision of dental care. In the second wave of the pandemic, fear and anxiety still exist due to the continued presence of the pandemic, the emergence of new variants with different patterns of infectivity and the cumulative health, social and economic impacts. Our findings provide an analysis of how various factors were associated with practice closure in the first wave and how they may shape countries’ or individuals’ decisions to close their practices in the second wave or in future crisis situations. The need for packages to support the profession and for programs to maintain oral health for the public assumes greater importance as the pandemic continues. This high level of practice closure seen in the first wave seems to be unrealistic and may be unneeded for future scenarios.

75% of the dentists who participated in this study reported practice closure. International guidelines on the provision of dental care during the pandemic vary from country to country. For instance, China only allows public dental and general hospitals to deal with emergency cases [33], the USA, California, in particular, urges practitioners to close their clinics [34], the UK prescribes decreasing the number of examined patients [35], and some countries offer no guidance whatsoever [36]. The frequency of dental practice closure in the present study was higher than that reported for other, non- dental specialties in a WHO survey of 155 countries where 53% of participating countries reported disruption of treatment for hypertension, 49% for diabetes, 42% for cancer and 31% for cardiovascular emergencies [37]. However, the frequency of closure observed in this study was similar to that reported in the USA, where 79% of all dental practices except for those providing emergency care, were closed [38]. The impact of practice closure and the suspension of dental care on oral health is yet to be quantified.

In the present study, fear of income reduction because of COVID-19 was among the three top fears reported by dentists and fear was associated with practice closure. This agrees with reports showing that there was lower patient volume due to avoidance of healthcare facilities and fear of COVID-19 which resulted in financial losses in dental practices and reduced ability to pay employees. A US survey conducted in March 2020 reported that 28% of dentists were unable to pay their staff and 45% made partial payments [39, 40]. It was estimated that if the current lockdown continued, a large proportion of dentists and dental practices will face serious financial hardships [38]. A British survey also reported increasing risks of permanent closure of dental practices, especially in the primary care sector, as the pandemic continues with greater risks in the absence of support measures such as loans [41].

The financial crisis brought about by COVID-19 is not likely to end in the coming period, and a potentially massive impact on the dental profession may be expected. The present study showed that fear of infection is one of the factors associated with practice closure among dentists in the private sector. This finding agrees with a study reporting that a high level of anxiety is associated with more dentists indicating a desire to close their practice [42].

In the present study, dentists in the academia were more likely to report practice closure. This finding agrees with previous data from North America which indicated that dental care in teaching clinics was suspended and only emergency treatment was offered [39]. The present study also showed that dentists working in hospitals were less likely to report practice closure. This finding may be attributed to the high level of preparedness of hospitals. For example, hospitals are more likely than other healthcare facilities to be equipped with high-level PPE for protection against aerosol-generating dental procedures [43, 44] and have strict infection control measures and more dental units to meet patients’ needs for emergency dental services [36, 45, 46].

This study showed fewer closures in group practices and more closures in solo practices. Group practices may be more resilient than solo practices in times of financial hardship because the former are more likely to have reserves and can afford to pool resources to bridge crises. Compared with larger practices, small-scale health care providers tend to be less profitable and are more vulnerable to financial threats [47].

The current study showed that practices in rural areas are more likely to close than urban practices if they are in the private sector but less likely to close if they are in the non-private sector. Rural healthcare facilities in the private sector usually operate on thin profit margins and have a small number of staff, which puts them at greater risk of closure compared to urban facilities to reduce financial and infection risks [48–50]. Consequently, rural practices in the non-private sector may be the only type of facility available to provide care for the local population, which could explain their lower likelihood of closure.

The present study showed that practice closure is also associated with country-level determinants. More hospital beds were associated with fewer closures in the non-private sector than in the private sector. Compared with less affluent countries, countries with high- resources and well-prepared healthcare systems are more likely to have better capacity to manage COVID-19 complications, resulting in lower mortality rates, panic, and anxiety as well as less chances of dental practice closure [51, 52]. This study found fewer private practice closures in HICs than in non-HICs. This finding agrees with reports that some HICs provide financial support for dental practices to avoid closure due to economic losses by offering funds, loans, and credit to help with the payment of salaries and supplies [40, 53–56]. In addition, dentists in HICs also generally have higher per capita income, which translates to better financial stability despite decreased revenues and reduced needs for practice closure. No such measures were reported in less- affluent countries, where no economic support plans were formulated to help the dental sector despite its needs.

This study has some limitations. First, its cross-sectional design cannot prove causality. Second, the convenience sample cannot support statistical representativeness. The study included a large number of dentists from many countries all over the world, with different professional backgrounds and healthcare system characteristics, and this increases the generalizability of findings. However, the number of participants per country varied widely and because of this and the convenience selection, the samples were not representative of the respective countries. We accounted for country level variation in the multilevel modeling but did not report estimates by countries for this reason. Also, some countries were under-represented, especially HIC, as the USA and some were not represented, such French speaking countries and China. We claim that traditional statistical representativeness cannot be achieved by random sampling for the present study targeting dentists in several countries for the following reasons: (1) not all countries have a comprehensive list/ archive of practicing dentists and (2) even in those where such records exist, the high non-response [57, 58] associated with COVID-19 studies would still pose a threat of selection bias. In addition, online surveys are known for their low response [59] which would yet add a further dimension for selection bias. The third limitation was because our study did not investigate the source of the decision to close dental practices and whether it was a political decision issued by governments or left to individual dentists’ choices. Fourth, we did not separately analyze complete closures and closures that allowed the management of emergency conditions and it would be useful to address this in future studies. Fifth, the dynamic nature of the pandemic and its spread were associated with changes in its knowledge base: some information that were believed to be correct in the initial stages may have failed the test of time and later became obsolete. It is important to consider the knowledge level of participants reported in this study within its time frame. The study estimated the frequency of practice closure which is important to assess the pandemic impact on oral health with implications for dental practice. Providing support to dentists in the private sector may help retain skilled personnel and reduce the devastating impact of the pandemic on dental services. Future studies are needed to assess the long-term impact of practice closure on the financial, psychological, and professional outcomes of dentists.

## Conclusions

COVID-19 had a considerable impact on dental practices around the world. During the first wave, most dentists reported practice closure because of COVID-19 with greater impact in the private sector than in the non-private sector. Personal, professional, and country-level factors were associated with practice closure. These findings help provide a profile of dentists with practices at greater risk of closure to plan appropriate support packages.

## Supplementary Information

**Additional file 1.** Questionnaire.

**Additional file 2.** Countries participating in the study.

**Additional file 3.** Italian version: Paura, minacce e conoscenza di COVID-19 per i dentisti. German version: Angst, Bedrohung und Kenntnis von COVID-19 für Zahnärzte.

## Data Availability

The dataset used in the current study is available from the corresponding author.

## Abbreviations

PPE: personal protective equipment
WHO: World Health Organization
HICs: high income countries
GNI: gross national income
UMICs: upper middle-income countries
LMICs: lower middle-income countries
LICs: lower income countries
CIs: confidence intervals
SPSS: statistical package for social sciences
SD: standard deviation
OR: odds ratio
