# Supplementary material for: Dental practice closure during the first wave of COVID-19 and associated professional, practice and structural determinants: a multi-country survey
Source: BMC Oral Health. 2021 May 7;21:243. doi: 10.1186/s12903-021-01601-4 (PMC8102846; doi:10.1186/s12903-021-01601-4)
Supplement: Supplementary file 1 — Additional file 1. Questionnaire. [file 12903_2021_1601_MOESM1_ESM.docx]

**Additional file 1**

**Questionnaire**

**Fear, threats and knowledge of COVID-19 for dental practitioners**

This questionnaire is for assessing dentists' responses to the COVID-19 outbreak. Kindly select answers that represent you the most. Please notice that your responses will be confidential and cannot be traced back to you. Only the research team will have access to the data you provide.

**Demographics**

1. **Age**:

20-30

31-40

41-50

51-60

61+

1. **Gender:**

Male

Female

1. **Country of practice: ……..**
2. **Specialty:**

Non specialist

Dental Anesthesiology

Dental Public Health

Endodontics

Oral and Maxillofacial Pathology

Oral and Maxillofacial Radiology

Oral and Maxillofacial Surgery

Orthodontics and Dentofacial Orthopedics

Pediatric Dentistry

Periodontics

Prosthodontics

1. **Nature of practice:**

Private sector

Governmental sector

Academic sector

1. **Type of practice:**

Solo “A clinic where single dentist practices”

Group “A dental center including more than one dentist”

Hospital

Other (please specify):

1. **Setting of practice:**

Urban

Rural

**Fear and threats of COVID-19, for each of the following statements indicate your agreement ranging from strongly agree, agree, uncertain, disagree or strongly disagree**

1. I am afraid of working in places where patients suspected of COVID-19 infection are treated.
   1. Strongly agree
   2. Agree
   3. Uncertain
   4. Disagree
   5. Strongly disagree
2. I am afraid of caring for patients infected with/ suspected of COVID-19.
   1. Strongly agree
   2. Agree
   3. Uncertain
   4. Disagree
   5. Strongly disagree
3. In spite of personal protective equipment (PPE) and infection prevention precautions, the risk of COVID-19 infection is high among health care personnel (HCP).
   1. Strongly agree
   2. Agree
   3. Uncertain
   4. Disagree
   5. Strongly disagree
4. Equipment and facilities required to protect HCP from COVID-19 infection are not adequately provided in healthcare facilities.
   1. Strongly agree
   2. Agree
   3. Uncertain
   4. Disagree
   5. Strongly disagree
5. HCP should get higher pay when treating patients infected with/ suspected of COVID-19 infection.
   1. Strongly agree
   2. Agree
   3. Uncertain
   4. Disagree
   5. Strongly disagree
6. I am afraid that a family member may be affected by COVID-19 infection.
   1. Strongly agree
   2. Agree
   3. Uncertain
   4. Disagree
   5. Strongly disagree
7. I am worried that my patient will not be receiving adequate care because of the outbreak.
   1. Strongly agree
   2. Agree
   3. Uncertain
   4. Disagree
   5. Strongly disagree
8. I am worried that my practice income would be affected because of the outbreak.
   1. Strongly agree
   2. Agree
   3. Uncertain
   4. Disagree
   5. Strongly disagree

**Read the following statements carefully and select the proper answer that reflect your knowledge about COVID-19**

1. The symptoms of COVID-19 infection are dry cough, fatigue and fever.
2. Yes*
3. No
4. I don’t know
5. To diagnose COVID-19 infection, a sample of upper and lower airways secretions is submitted for polymerase chain reaction examination.
6. Yes*
7. No
8. I don’t know
9. COVID-19 virus can survive on environmental surfaces.
10. Yes*
11. No
12. I don’t know
13. The incubation period of COVID-19 virus is between 2-14 days.
14. Yes*
15. No
16. I don’t know
17. COVID-19 virus can be transmitted through direct contact with respiratory tract secretions
18. Yes*
19. No
20. I don’t know
21. Hand hygiene prevents the transmission of COVID-19 infection.
22. Yes*
23. No
24. I don’t know
25. Environmental surfaces should be cleaned by diluted 10% bleaching solution.
26. Yes*
27. No
28. I don’t know
29. Droplet, contact and airborne precautions should be followed when dealing with patients suspected of/ infected with COVID-19.
30. Yes*
31. No
32. I don’t know
33. Preoperative 1% hydrogen peroxide mouth rinse should be used before dental procedures for patients suspected of/ infected with COVID-19.
34. Yes*
35. No
36. I don’t know
37. Extraoral dental radiographies are appropriate alternatives during COVID-19 outbreak.
38. Yes*
39. No
40. I don’t know
41. Social distancing and removing all reading sources and toys in dental clinic should be considered.
42. Yes*
43. No
44. I don’t know
45. Providing task-specific education and training on preventing transmission of infectious agents for front line staff should be considered.
46. Yes*
47. No
48. I don’t know
49. Elective dental treatment should be postponed.
50. Yes*
51. No
52. I don’t know
53. Rubber dam and high rapid suction is mandatory while treating all patients during outbreak.
54. Yes*
55. No
56. I don’t know
57. Urgent dental care treatments, should be treated as minimally invasively as possible.
58. Yes*
59. No
60. I don’t know

**Practice closed because of the outbreak**: yes/no
