# Supplementary material for: Dental practice closure during the first wave of COVID-19 and associated professional, practice and structural determinants: a multi-country survey
Source: BMC Oral Health. 2021 May 7;21:243. doi: 10.1186/s12903-021-01601-4 (PMC8102846; doi:10.1186/s12903-021-01601-4)
Supplement: Supplementary file 2 — Additional file 2. Countries participating in the study. [file 12903_2021_1601_MOESM2_ESM.docx]

**Additional file 2**

Countries participating in the study

| **Country** | **N** | **%** |
| --- | --- | --- |
| 1. Albania | 3 | 0.1 |
| 1. Australia | 4 | 0.1 |
| 1. Denmark | 30 | 0.9 |
| 1. Egypt | 771 | 23.6 |
| 1. Germany | 18 | 0.6 |
| 1. Hungary | 3 | 0.1 |
| 1. India | 253 | 7.7 |
| 1. Indonesia | 440 | 13.4 |
| 1. Iraq | 4 | 0.1 |
| 1. Italy | 146 | 4.5 |
| 1. KSA | 232 | 7.1 |
| 1. Lebanon | 4 | 0.1 |
| 1. Malaysia | 109 | 3.3 |
| 1. Mexico | 3 | 0.1 |
| 1. Myanmar | 543 | 16.6 |
| 1. Nepal | 5 | 0.2 |
| 1. Netherlands | 4 | 0.1 |
| 1. Pakistan | 42 | 1.3 |
| 1. Palestine | 4 | 0.1 |
| 1. Qatar | 13 | 0.4 |
| 1. Romania | 3 | 0.1 |
| 1. Serbia | 3 | 0.1 |
| 1. Syria | 3 | 0.1 |
| 1. Tanzania | 3 | 0.1 |
| 1. Turkey | 3 | 0.1 |
| 1. UAE | 27 | 0.8 |
| 1. UK | 410 | 12.5 |
| 1. USA | 30 | 0.9 |
| 1. Yemen | 130 | 4.0 |
| **Total** | **3243** | **100.0** |
