# Supplementary material for: Dental practice closure during the first wave of COVID-19 and associated professional, practice and structural determinants: a multi-country survey
Source: BMC Oral Health. 2021 May 7;21:243. doi: 10.1186/s12903-021-01601-4 (PMC8102846; doi:10.1186/s12903-021-01601-4)
Supplement: Supplementary file 3 — Additional file 3. Italian version: Paura, minacce e conoscenza di COVID-19 per i dentisti. German version: Angst, Bedrohung und Kenntnis von COVID-19 für Zahnärzte. [file 12903_2021_1601_MOESM3_ESM.docx]

**Italian version**

**Paura, minacce e conoscenza di COVID-19 per i dentisti**

Questo questionario serve a valutare le risposte dei dentisti allo scoppio di COVID-19. Seleziona le risposte che ti rappresentano di più. Tieni presente che le tue risposte saranno riservate e non potranno essere ricondotte a te. Solo il team di ricerca avrà accesso ai dati forniti.

**I. Demografia**

**1.Età**

**2.Sesso**

Maschio

Femmina

**3.Paese di pratica**

**4.Specialità:**

Non specialista

Anestesiologia dentale

Salute pubblica dentale

Endodonzia

Patologia orale e maxillofacciale

Radiologia orale e maxillofacciale

Chirurgia orale e maxillofacciale

Ortodonzia e ortopedia dentofacciale

Odontoiatria Pediatrica

Parodontologia

Protesi

**5.Natura della pratica:**

Settore privato

Settore governativo

Settore accademico

**6.Tipo di pratica:**

Solo "Una clinica dove si esercita il singolo dentista"

Gruppo "Un centro odontoiatrico che comprende più di un dentista"

Ospedale

Altro (specificare):

**7.Impostazione della pratica:**

Urbano

Rurale

**8.Esercizio chiuso a causa dell'epidemia:** sì / no

**II. Paura e minacce di COVID-19, per ognuna delle seguenti affermazioni esprimi il tuo grado di accordo da fortemente d'accordo, d'accordo, incerto, in disaccordo o fortemente in disaccordo.**

**1.Ho paura di lavorare in luoghi in cui vengono trattati pazienti sospettati di infezione da COVID-19.**

1. Fortemente d’accordo
2. D’accordo
3. Incerto
4. In disaccordo
5. Fortemente in disaccordo

**2.Ho paura di prendermi cura dei pazienti infetti / sospettati di COVID-19.**

1. Fortemente d’accordo
2. D’accordo
3. Incerto
4. In disaccordo
5. Fortemente in disaccordo

**3.Nonostante i dispositivi di protezione individuale (DPI) e le precauzioni di prevenzione delle infezioni, il rischio d’infezione da COVID-19 è elevato tra il personale sanitario.**

1. Fortemente d’accordo
2. D’accordo
3. Incerto
4. In disaccordo
5. Fortemente in disaccordo

**4.Le attrezzature e le strutture necessarie per proteggere il personale sanitario dall'infezione COVID-19 non sono adeguatamente fornite nelle strutture sanitarie.**

1. Fortemente d’accordo
2. D’accordo
3. Incerto
4. In disaccordo
5. Fortemente in disaccordo

**5.L'operatore sanitario dovrebbe ottenere una retribuzione più elevata nel trattamento di pazienti infetti / sospettati di infezione da COVID-19.**

1. Fortemente d’accordo
2. D’accordo
3. Incerto
4. In disaccordo
5. Fortemente in disaccordo

**6.Temo che un membro della famiglia possa essere colpito dall'infezione COVID-19.**

1. Fortemente d’accordo
2. D’accordo
3. Incerto
4. In disaccordo
5. Fortemente in disaccordo

**7.Sono preoccupato che il mio paziente non riceverà cure adeguate a causa dell'epidemia.**

1. Fortemente d’accordo
2. D’accordo
3. Incerto
4. In disaccordo
5. Fortemente in disaccordo

**8.Sono preoccupato che il mio reddito sia influenzato a causa dell'epidemia.**

1. Fortemente d’accordo
2. D’accordo
3. Incerto
4. In disaccordo
5. Fortemente in disaccordo

**III. Leggi attentamente le seguenti dichiarazioni e seleziona la risposta corretta che riflette la tua conoscenza di COVID-19**

**1. I sintomi dell'infezione da COVID-19 sono tosse secca, affaticamento e febbre.**

a.sì

b.No

c.Non lo so

**2.Per diagnosticare l'infezione da COVID-19, viene inviato un campione di secrezioni delle vie aeree superiori e inferiori per l'esame della reazione a catena della polimerasi.**

a.sì

b.No

c.Non lo so

**3.Il virus COVID-19 può sopravvivere su superfici ambientali.**

a.sì

b.No

c.Non lo so

**4.Il periodo di incubazione del virus COVID-19 è tra 2-14 giorni.**

a.sì

b.No

c.Non lo so

**5.Il virus COVID-19 può essere trasmesso attraverso il contatto diretto con le secrezioni del tratto respiratorio**

a.sì

b.No

c.Non lo so

**6.L'igiene delle mani impedisce la trasmissione dell'infezione COVID-19.**

a.sì

b.No

c.Non lo so

**7.Le superfici ambientali devono essere pulite con una soluzione di candeggio diluita al 10%.**

a.sì

b.No

c.Non lo so

**8.Precauzioni per la trasmissione aerea, da contatto e attraverso gocciolone (droplets) devono essere seguite quando si tratta di pazienti sospettati / infetti da COVID-19.**

a.sì

b.No

c.Non lo so

**9.Il risciacquo orale preoperatorio all'1% di perossido di idrogeno deve essere usato prima di procedure dentali per pazienti sospettati / infetti da COVID-19.**

a.sì

b.No

c.Non lo so

**10.Le radiografie dentali extraorali sono alternative appropriate durante l'epidemia di COVID-19.**

a.sì

b.No

c.Non lo so

**11.Devono essere prese in considerazione la distanza sociale e la rimozione di tutte le fonti di lettura e giocattoli nella clinica dentale.**

a.sì

b.No

c.Non lo so

**12.Dovrebbe essere presa in considerazione la possibilità di fornire istruzione e formazione specifiche per compiti sulla prevenzione della trasmissione di agenti infettivi per il personale di prima linea.**

a.sì

b.No

c.Non lo so

**13.Il trattamento dentale elettivo dovrebbe essere posticipato.**

a.sì

b.No

c.Non lo so

**14.La diga di gomma e l'aspirazione rapida elevata sono obbligatorie durante il trattamento di tutti i pazienti durante l'epidemia.**

a.sì

b.No

c.Non lo so

**15.I trattamenti di cure odontoiatriche urgenti devono essere trattati nel modo meno invasivo possibile.**

a.sì

b.No

c.Non lo so

**German version**

**Angst, Bedrohung und Kenntnis von COVID-19 für Zahnärzte**

Dieser Fragebogen dient zur Bewertung der Reaktionen von Zahnärzten auf den COVID-19-Ausbruch. Bitte wählen Sie sorgfältig die Antworten aus, die Sie am meisten ansprechen. Bitte beachten Sie, dass Ihre Antworten vertraulich sind und nicht auf Sie zurückgeführt wurden können. Nur das Forschungsteam hat Zugriff auf die von Ihnen bereitgestellten Daten.

1. **Demografie:**
2. **Alter:**
3. (20-30)
4. (31-40)
5. (41-50)
6. (51-60)
7. (61+)

**2. Geschlecht:**

1. Männlich
2. Weiblich

**3. Land:**

1. Ägypten
2. Saudi-Arabien
3. Vereinigte Arabische Emirate
4. Kuwait
5. Katar
6. Deutschland
7. Großbritannien
8. Die USA
9. Austria
10. Dänemark
11. Italien
12. Anderes(bitte angeben):

**4**. **Spezialgebiet:**

1. Nicht spezialisiert
2. Zahn Anästhesiologie
3. Öffentliche Zahngesundheit
4. Endodontie
5. Orale und maxillofaziale Pathologie
6. Orale und maxillofaziale Radiologie
7. Mund- und Kieferchirurgie
8. Kieferorthopädie und dentofaziale Orthopädie
9. Kinderzahnheilkunde
10. Parodontologie
11. Prothetik

**5. Sektorzugehörigkeit:**

1. Privater Bereich
2. Regierungssektor
3. Akademischer Sektor

**6. Art der Praxis:**

1. Einzelpraxis
2. Gruppe „Ein Zahnarztzentrum mit mehr als einem Zahnarzt“
3. Krankenhaus
4. Sonstiges (bitte angeben):

**7. Standort:**

1. Städtisch
2. Ländlich

**8. Praxis wegen des Ausbruchs geschlossen:**

1. Ja
2. nein
3. **Ängste und Drohungen vor COVID-19, für jede der folgenden Aussagen geben Sie eine Stellungnahme.**
4. Ich habe Angst, an Orten zu arbeiten, an denen Patienten mit Verdacht auf COVID-19-Infektion behandelt werden. (Ich habe Angst vor das arbeiten an Orten wo patienten mit Verdacht auf COVID Infektion behandelt werden.
5. trifft voll zu
6. trifft eher zu
7. trifft teils, teils zu
8. trifft eher nicht zu
9. trifft nicht zu
10. Ich habe Angst, mich um Patienten zu kümmern, die mit COVID-19 infiziert sind oder im Verdacht stehen.
    1. trifft voll zu
    2. trifft eher zu
    3. trifft teils, teils zu
    4. trifft eher nicht zu
    5. trifft nicht zu
11. Trotz persönlicher Schutzausrüstung (PSA) und Vorsichtsmaßnahmen zur Infektionsprävention ist das Risiko einer COVID-19-Infektion beim Gesundheitspersonal (HCP) hoch.
    1. trifft voll zu
    2. trifft eher zu
    3. trifft teils, teils zu
    4. trifft eher nicht zu
    5. trifft nicht zu
12. Ausrüstung und Einrichtungen, die zum Schutz von Gesundheitspersonal vor COVID-19-Infektionen erforderlich sind, werden in Gesundheitseinrichtungen nicht ausreichend bereitgestellt.
    1. trifft voll zu
    2. trifft eher zu
    3. trifft teils, teils zu
    4. trifft eher nicht zu
    5. trifft nicht zu
13. Medizinische Fachkräfte sollte bei der Behandlung von Patienten, die mit COVID-19-Infektion infiziert sind oder unter Verdacht stehen, eine höhere Bezahlung erhalten.
    1. trifft voll zu
    2. trifft eher zu
    3. trifft teils, teils zu
    4. trifft eher nicht zu
    5. trifft nicht zu
14. Ich habe angst, dass einen meines Familienmitglied COVID-19- Infektion erwerben könnte.
    1. trifft voll zu
    2. trifft eher zu
    3. trifft teils, teils zu
    4. trifft eher nicht zu
    5. trifft nicht zu
15. Ich mache mir Sorgen, dass meine Patienten wegen des Ausbruchs nicht ausreichend versorgt werden.
    1. trifft voll zu
    2. trifft eher zu
    3. trifft teils, teils zu
    4. trifft eher nicht zu
    5. trifft nicht zu
16. Ich mache mir Sorgen, dass Einkommen der Praxis durch den Ausbruch beeinträchtigt wird.
    1. trifft voll zu
    2. trifft eher zu
    3. trifft teils, teils zu
    4. trifft eher nicht zu
    5. trifft nicht zu
17. **Lesen Sie die folgenden Aussagen sorgfältig durch und wählen Sie die richtigevAntwort aus, die Ihr Wissen über COVID-19 widerspiegelt**
18. Die Symptome einer COVID-19-Infektion sind trockener Husten, Müdigkeit und Fieber.
19. ja
20. nein
21. ich weiß es nicht
22. Zur Diagnose einer COVID-19-Infektion wird eine Probe der Sekrete der oberen und unteren Atemwege zur Untersuchung der Polymerasekettenreaktion vorgelegt.
    1. ja
    2. nein
    3. ich weiß es nicht
23. Das COVID-19-Virus kann auf Oberflächen bei gewöhnlicher Umwelt überleben.
    1. ja
    2. nein
    3. ich weiß es nicht
24. Die Inkubationszeit des COVID-19-Virus liegt zwischen 2 und 14 Tagen.
    1. ja
    2. nein
    3. ich weiß es nicht
25. Das COVID-19-Virus kann durch direkten Kontakt mit Sekreten der Atemwege übertragen werden
    1. ja
    2. nein
    3. ich weiß es nicht
26. Händehygiene verhindert die Übertragung einer COVID-19-Infektion.
    1. ja
    2. nein
    3. ich weiß es nicht
27. Oberflächen sollten mit verdünnter 10% iger Bleichlösung gereinigt werden.
    1. ja
    2. nein
    3. ich weiß es nicht
28. Bei Patienten, bei denen der Verdacht auf COVID-19 besteht oder die mit COVID-19 infiziert sind, sollten Vorsichtsmaßnahmen gegen Tröpfchen, Kontakt und Luft getroffen werden.
    1. ja
    2. nein
    3. ich weiß es nicht
29. Präoperative 1% ige Wasserstoffperoxid-Mundspülung sollte vor zahnärztlichen Eingriffen bei Patienten angewendet werden, bei denen der Verdacht auf / die Infektion mit COVID-19 besteht.
    1. ja
    2. nein
    3. ich weiß es nicht
30. xtraorale zahnärztliche Röntgenaufnahmen sind geeignete Alternativen während des COVID-19-Ausbruchs.
    1. ja
    2. nein
    3. ich weiß es nicht
31. Soziale Distanzierung und das Entfernen aller Zeitschriften und Spielzeuge in der Zahnklinik sollten berücksichtigt werden.
    1. ja
    2. nein
    3. ich weiß es nicht
32. Die Bereitstellung einer aufgabenspezifischen Schulung und Schulung zur Verhinderung der Übertragung von Infektionserregern für Mitarbeiter an vorderster Front sollte in Betracht gezogen werden.
    1. ja
    2. nein
    3. ich weiß es nicht
33. Die selektive Zahnbehandlung sollte verschoben werden.
    1. ja
    2. nein
    3. ich weiß es nicht
34. Kofferdam und hohe schnelle Absaugung sind bei der Behandlung aller Patienten während des Ausbruchs obligatorisch.
    1. ja
    2. nein
    3. ich weiß es nicht

17. Dringende Behandlungen sollten so minimalinvasiv wie möglich behandelt werden.

1. ja
2. nein
3. ich weiß es nicht
